# A newly-developed community microarray resource for transcriptome profiling in *Brassica *species enables the confirmation of *Brassica*-specific expressed sequences

**DOI:** 10.1186/1471-2229-9-50

**Published:** 2009-05-08

**Authors:** Martin Trick, Foo Cheung, Nizar Drou, Fiona Fraser, Edward K Lobenhofer, Patrick Hurban, Andreas Magusin, Christopher D Town, Ian Bancroft

**Affiliations:** 1 John Innes Centre, Norwich Research Park, Colney, Norwich, NR4 7UH, UK; 2 The J Craig Venter Institute, 9704 Medical Center Drive, Rockville, MD 20850, USA; 3 Cogenics, A Division of Clinical Data, Inc, 100 Perimeter Park Drive, Suite C, Morrisville, NC 27560, USA; 4 Current address : Amgen Inc, 1 Amgen Center Drive, Thousand Oaks, CA 91320, USA

## Abstract

**Background:**

The *Brassica *species include an important group of crops and provide opportunities for studying the evolutionary consequences of polyploidy. They are related to *Arabidopsis thaliana*, for which the first complete plant genome sequence was obtained and their genomes show extensive, although imperfect, conserved synteny with that of *A. thaliana*. A large number of EST sequences, derived from a range of different *Brassica *species, are available in the public database, but no public microarray resource has so far been developed for these species.

**Results:**

We assembled unigenes using ~800,000 EST sequences, mainly from three species: *B. napus*, *B. rapa *and *B. oleracea*. The assembly was conducted with the aim of co-assembling ESTs of orthologous genes (including homoeologous pairs of genes in *B. napus *from each of the A and C genomes), but resolving assemblies of paralogous, or paleo-homoeologous, genes (*i.e*. the genes related by the ancestral genome triplication observed in diploid *Brassica *species). 90,864 unique sequence assemblies were developed. These were incorporated into the BAC sequence annotation for the *Brassica rapa *Genome Sequencing Project, enabling the identification of cognate genomic sequences for a proportion of them. A 60-mer oligo microarray comprising 94,558 probes was developed using the unigene sequences. Gene expression was analysed in reciprocal resynthesised *B. napus *lines and the *B. oleracea *and *B. rapa *lines used to produce them. The analysis showed that significant expression could consistently be detected in leaf tissue for 35,386 unigenes. Expression was detected across all four genotypes for 27,355 unigenes, genome-specific expression patterns were observed for 7,851 unigenes and 180 unigenes displayed other classes of expression pattern. Principal component analysis (PCA) clearly resolved the individual microarray datasets for *B. rapa*, *B. oleracea *and resynthesised *B. napus*. Quantitative differences in expression were observed between the resynthesised *B. napus *lines for 98 unigenes, most of which could be classified into non-additive expression patterns, including 17 that showed cytoplasm-specific patterns. We further characterized the unigenes for which A genome-specific expression was observed and cognate genomic sequences could be identified. Ten of these unigenes were found to be *Brassica*-specific sequences, including two that originate from complex loci comprising gene clusters.

**Conclusion:**

We succeeded in developing a *Brassica *community microarray resource. Although expression can be measured for the majority of unigenes across species, there were numerous probes that reported in a genome-specific manner. We anticipate that some proportion of these will represent species-specific transcripts and the remainder will be the consequence of variation of sequences within the regions represented by the array probes. Our studies demonstrated that the datasets obtained from the arrays can be used for typical analyses, including PCA and the analysis of differential expression. We have also demonstrated that *Brassica*-specific transcripts identified *in silico *in the sequence assembly of public EST database accessions are indeed reported by the array. These would not be detectable using arrays designed using *A. thaliana *sequences.

## Background

The cultivated *Brassica *species are the group of crops most closely related to *Arabidopsis thaliana*. They are members of the Brassicaceae (sometimes referred to as the Crucifereae) family [1]. The species typically termed the "diploid" *Brassica *species, *B. rapa *(*n *= 10), *B. nigra *(*n *= 8) and *B. oleracea *(*n *= 9) contain the A, B and C genomes, respectively. Each pairwise combination has hybridized spontaneously to form the three allotetraploid species [2], *B. napus *(*n *= 19, comprising A and C genomes), *B. juncea *(*n *= 18, comprising A and B genomes) and *B. carinata *(*n *= 17, comprising B and C genomes). The genome of *B. rapa *is the smallest, at *ca*. 500 Mb [3], and a genome sequencing project is under way, with both sequences and sequence annotations in the public domain 

The lineages of *B. rapa *and *B. oleracea *diverged *ca*. 3.7 Mya [4] and genetic mapping has confirmed that the overall organisation of their genomes is highly collinear [5]. Their hybridisation to form *B. napus *probably occurred during human cultivation, *i.e*. less than 10,000 years ago. Comparative genetic mapping showed that the progenitor A and C genomes in *B. napus *have undergone little or no gross rearrangement during that time [6] and also revealed extensive duplication within the *Brassica *genomes [5]. Recent cytogenetic studies have shown that a distinctive feature of the *Brassiceae *tribe, of which the *Brassica *species are members, is that they contain extensively triplicated genomes [7].

Even at the resolution of linkage maps, extensive collinearity can be identified between the genomes of *Brassica *species and *A. thaliana*. For example, a landmark study using sequenced RFLP markers demonstrated that 21 segments of the genome of *A. thaliana*, representing almost its entirety, could be replicated and rearranged to generate a structure approximating that of the *B. napus *genome [8]. A study across the Brassicaceae subsequently identified 24 conserved chromosomal blocks, relating them to a proposed ancestral karyotype of *n *= 8 [9]. A number of genome analyses have been conducted in *B. oleracea, B. rapa *and *B. napus *using physical mapping techniques. The results have shown that the diploid *Brassica *genomes contain extensive triplication, consistent with their having evolved from a hexaploid ancestor [10-12]. Two sequence-level studies, one in *B. oleracea *[13] and one in *B. rapa *[14] have provided further support for the hypothesis of hexaploid ancestry for the *Brassica *species. If this hypothesis were true, the duplicate genes we observe in the extant diploid genomes would formally be "paleo-homoeologues". However, here we will use the more general term paralogue, which is free of this assumption, to clearly delineate from the recognisable homoeologues in *B. napus *arising from the very recent hybridisation of the A and C genomes. The studies using physical mapping and sequencing approaches showed that, although sets of three related genome segments (paralogues) will often be identifiable within the genome of the diploid *Brassica *species, a proportion of the genes in these segments will have been lost.

*Brassica *polyploids can be synthesised artificially. For example, *B. napus *can be resynthesised by hybridization of *B. rapa *and *B. oleracea*. However, it has been found that such lines display genome instability [15], which can persist for many generations and is thought to involve homoeologous non-reciprocal translocations. They have been shown to be correlated with qualitative changes in the expression of specific genes and with phenotypic variation [16].

Microarrays have become a widely-used tool for transcriptome analysis in plants. Essentially, they consist of an immobilised array of DNA sequences (probes) which are hybridized *in situ *using fluorescently-labelled sequences (targets) derived by reverse transcription of polyadenylated transcripts. Imaging of the hybridized array, followed by computational analysis of the signal intensity data, leads to a quantification of the transcript abundance, in the sampled tissue, of the genes represented by the probes in the array. There are numerous microarray platforms available and they have been applied to a wide range of studies in plant biology, reviewed by Galbraith [17].

As the *Brassica *species diverged from *A. thaliana *only *ca*. 17 Mya [18], exon sequences show a high level of conservation, *ca*. 85% at the nucleotide level [19]. Therefore some types of microarrays designed for use in *A. thaliana *can be used for the analysis in *Brassica *of the related genes. However, an analysis of *ca*. 100,000 *Brassica *EST sequences showed that *ca*. 9% showed no similarity with any gene in *A. thaliana *[14]. *A. thaliana*-based microarrays therefore would fail to measure the expression of a significant number of *Brassica *genes. In addition, *Brassica *genomes show extensive triplication, with the sub-genomes estimated to have diverged *ca*. 14 Mya [13,14,18]. *A. thaliana*-based microarrays would lack the capability to resolve the contributions to the transcriptome of such families of paralogous genes. Consequently, a number of groups have developed *Brassica *cDNA-based microarrays, but these have been based upon relatively modest EST collections and none are available as community resources. We aimed to address this deficiency by developing a microarray based upon all public EST data, validating its utility for transcriptome analysis across multiple Brassica species, and placing it in the public domain. The validation experiment involved transcriptome analysis in two "resynthesised" *B. napus *lines and their *B. rapa *and *B. oleracea *progenitors. This experimental design enables the identification of both species-specific and genome-specific expression, whilst the long oligonucleotides used essentially eliminate the possible complications due to allelic variation (SNPs and small indels).

## Results

### Assembly of Brassica unigenes

All available *Brassica *species ESTs were downloaded from GenBank in September, 2007. These consisted of three principal sets: *B. napus *(567,240), *B. rapa *(180,611) and *B. oleracea *(59,696). A total of 810,254 ESTs after cleaning and removal of low quality and short (<100 bp) sequences was reduced to 803,326 reads. Since the initial goal was to develop a widely useful *Brassica *microarray, all available ESTs were assembled together using the TGICL software package [20] with default settings (94% identity, 90% coverage). The statistics for this assembly are shown in Table 1. Sequences were oriented either based on their alignment with a known protein or by the presence of a polyA (polyT) tail. A total of 3,694 sequences (330 assemblies and 3364 singletons) could not be oriented and were thus represented in both orientations in the data set from which the array was designed, making 94,558 sequences in all. The assemblies and singletons were annotated by searching against NCBI Uniprot100 using a cut-off of 1E-5. A total of 72,148 sequences were annotated.

**Table 1:** Summary statistics of unigene assembly

Total number of reads	803,326
Total unique sequences	90,864

Total assemblies	42,642

ESTs in assemblies	751,410

Total singletons	48,222

Total base count	64,044,420 bp

Minimum length	101 bp

Average length	677 bp

Maximum length	3,786 bp

### Incorporation of assemblies into the Brassica genome sequence annotation

As partners in a multinational consortium to sequence the gene space of the *Brassica rapa *genome, we make available (from  a first-pass annotation of completed BACs immediately on deposition in the public sequence databases. The annotation is rendered through the GBrowse genome browser system [21]. For the present study, 673 BAC sequences were available for analysis and were annotated. The sequence coverage was approximately 80 Mbp, which is equivalent to ~14.5% coverage of the entire ~550 Mbp *B. rapa *genome *pro rata *[8], but this might represent a greater fraction of the gene space because the original seed BACs and hence the scaffold extensions were targeted to the gene-rich euchromatin.

There were 19,148 separate instances of unigenes aligning within this annotation set and 10,606 of the 17,862, (59.4%) FGENESH gene models predicted had EST support arising from some overlap with these EST alignments. Of the 90,864 unigenes comprising the assembly, 13,938 (15.4%) appeared at least once within the annotation set, including 38 of the unigenes represented in both orientations. Gene predictions around the latter may aid in their resolution.

### Design of the microarray

One of the primary requirements for the design of the microarray was that it should be applicable for transcriptome analysis across a range of *Brassica *species. Therefore, we required a platform based on "long oligonucleotide" probes in order to minimise susceptibility to SNP variation across species, whilst retaining the capability of resolving the transcripts of significantly diverged gene families, such as those with paralogous relationships within the *Brassica *genomes. To accommodate these design requirements, the Agilent Technologies microarray platform, which uses 60-mer oligonucleotide probes, was selected .

The assembled *Brassica *sequences (94,558 instances including those represented in both orientations) were submitted to Agilent Technologies' eArray web portal for gene expression probe design. For each 60-mer oligonucleotide probe that is designed using this tool, a base composition score is calculated to reflect the theoretical performance of the probe in standard hybridization conditions. Probes with a base composition score greater than or equal to 3 were omitted from the final design. This resulted in a total of 91,854 unique probes (including 6,989 derived from oppositely oriented pairs of sequences) that were included in the microarray design, of which 10,466 were predicted to have cross-hybridization potential. To utilize the full capacity of the microarray, 11,893 probes were randomly selected to be represented in duplicate in the final design, which also included Agilent Technologies' standard panel of quality control and spike-in probes. This design was then used to manufacture microarrays using Agilent Technologies' SurePrint™ Technology in the 2× 104 k format (two microarrays containing ~104,000 probes on a single 1" × 3" glass slide).

### Qualitative analysis of gene expression across genotypes

The experimental design used to test the performance of the microarray included four genotypes: two "resynthesized" *B. napus *lines and their progenitor *B. rapa *and *B. oleracea *lines. The nuclear genomes of the resynthesised *B. napus *lines should be identical but, as one (*B. napus *1) involved a cross of *B. oleracea *onto *B. rapa*, and the other (*B. napus *2) involved a cross of *B rapa *onto *B. oleracea*, they differ in cytoplasm, and hence contain different chloroplast and mitochondrial genomes. For each genotype, RNA was isolated from four biological replicates making a total of sixteen independent samples. The gene expression profile for each sample was generated by labelling and hybridizing each sample to one of 16 separate microarrays. The data are available from the GEO repository, accession number GSE15915.

**Figure 1:**
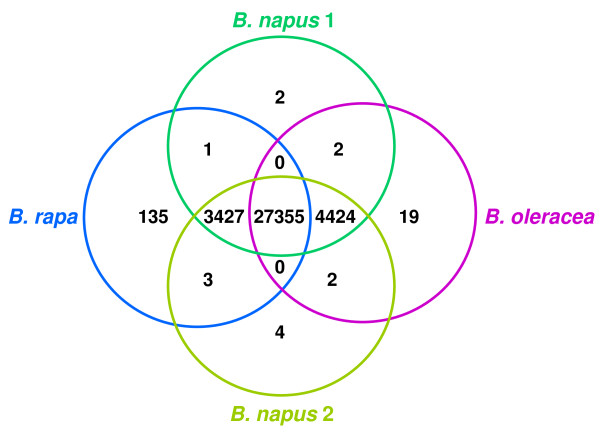
**Classification of qualitative expression patterns of unigenes**. Unigene classification by consistent, significant signals detected from each of the four genotypes analysed.

The parameters used for the assembly of the unigenes had been set such that transcribed sequences from orthologous genes, including homoeologues from the A and C genomes in *B. napus*, should co-assemble. In order to assess the number of probes that, nevertheless, report genome-specific expression, we used the presence or absence of significant signal (qualitative expression) for each probe to classify the expression pattern of the corresponding unigene. The probes were considered to give no signal if no significant expression was detected in any of the 16 microarrays. 31,705 of the 103,747 non-control probes on the array fell into this class. Of the probes for which significant expression was identified in at least one microarray, those that give only matching reports of either significant signal or no significant signal across every set of replicates (*i.e*. there were no instances of only 1, 2 or 3 replicate microarrays yielding significant signals from a particular genotype) were considered to have produced consistent reports of qualitative expression. In total, 39,689 probes produced consistent reports of qualitative expression and were used to classify qualitative expression patterns into 15 classes across the genotypes (see additional file 1: Spreadsheet1). The results, with duplicate probes removed in order to show the number of unigenes represented, are summarised in Figure 1. 1,109 of the 35,389 unigenes represented are from the dual-orientated subset, of which 108 were reported in both orientations. Significant qualitative expression can be detected across all genotypes for 27,355 unigenes. Genome-specific expression can be detected for 7,851 unigenes; 3,427 are expressed in *B. rapa *and *B. napus*, but not in *B. oleracea *and thus can be considered A genome-specific while by analogous criteria 4,424 can be considered C genome-specific. Significant expression was detected for 135 unigenes in *B. rapa *only and for 19 unigenes in *B. oleracea *only. No unigenes were expressed only in a diploid while 12 unigenes (not shown in Figure 1) were expressed only in a tetraploid. Very few unigenes (14 in total) were categorised into the remaining 9 classes of qualitative expression.

### Resolution of genotypes by Principal Component Analysis

**Figure 2:**
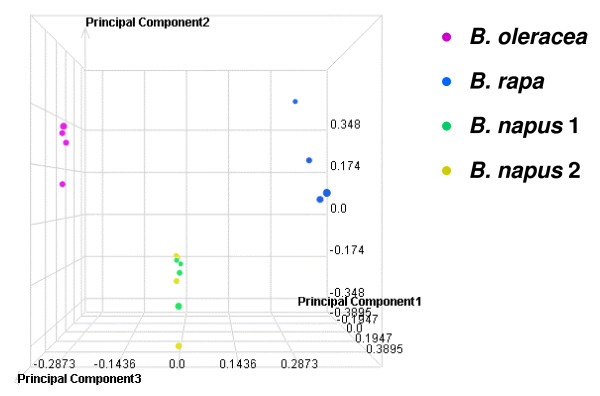
**Principal Component Analysis of gene expression in the four genotypes**. Microarray datasets for each of the individual samples subjected to analysis by three principal components. The proportions of the total variation explained by principal components 1, 2 and 3 are 22.1%, 13.6% and 10.1%, respectively.

In order to visualize the significant sources of variation within the entire data set, a principal component analysis (PCA) was performed. The PCA was performed using z-score transformed intensity measurements for all non-control probes on the microarray. The resulting scatterplot is depicted in Figure 2, with each colour representing a different genotype. The plot demonstrates that the biological replicates within each genotype cluster closely together. Furthermore, the largest source of variation in the gene expression data is the different species as evidenced by the distinct groupings of each genotype along the x-axis (which depicts principal component 1). There was limited resolution of the resynthesised *B. napus *lines, which differed only by cytoplasm.

### Identification of differential gene expression in resynthesised *B. napus*

Apart from heritable epigenetic differences, the nuclear genomes of the resynthesised *B. napus *lines should be identical, but their chloroplast and mitochondrial genomes differ. We investigated whether the microarray was capable of detecting any cytoplasm-specific differences in gene expression or any deviation from the expected additive contributions of the parental nuclear genomes to the transcriptome of the amphidiploid, typically termed transcriptome remodelling or non-additive gene expression. Quantitative expression was compared between the resynthesised *B. napus *lines. 98 unigenes were identified that showed significant (P < 0.001) expression differences between the two lines (see additional file 2: Spreadsheet2) For each of these unigenes, the genome of origin (nuclear, chloroplast or mitochondrion) was determined by using BLAST to identify similarity between the unigene sequence and annotated genes or other sequences in the public databases. The expression patterns were further classified, where possible, based upon significant differences between expression in other pairs of genotypes, *i.e*. involving the *B. oleracea *and *B. rapa *genotypes (see additional file 3: Spreadsheet3).

Seventeen unigenes showed cytoplasm-specific expression profiles (*i.e*. there is a significant difference between the reported expression in the *B. oleracea *and *B. rapa *lines and the expression reported in the resynthesised *B. napus *lines corresponds to that of the maternal parent in the respective hybridization). Of these, 12 unigenes are of chloroplast origin, two are of mitochondrial origin and three are of nuclear origin. These patterns are consistent with cytoplasmic inheritance (chloroplast and mitochondrial genes) or epigenetic imprinting (nuclear genes). Non-additive expression could be identified for 60 unigenes, 58 of which are nuclear-encoded and two that are mitochondrial. The expression patterns of 21 unigenes (13 nuclear-encoded, five chloroplast encoded and three mitochondrion-encoded) that showed significant differences in expression between the resynthesised *B. napus *lines could not be classified, as a result of lack of significance in expression levels between other combinations of genotypes. These results show that the expression data generated using the microarray are, with four biological replicates, of a sufficiently high quality to enable the classification of expression patterns for 77 of the 98 unigenes (79%) showing significant differences in expression between the resynthesised *B. napus *lines, including the identification of many cytoplasm-specific expression patterns for genes encoded by chloroplasts or mitochondria.

### Characterization of sequences showing genome-specific expression

Expression of 7,851 unigenes was found in both *B. napus *lines and only one or other of the two diploids. Of these, 3,427 are from the A genome. BLASTN was used to scan the sequenced BACs for these probes and for the corresponding complete unigene sequences. Of the aligned (cognate) unigenes, ten were randomly selected for further analysis. The entire unigene sequences were used to identify, using BLAST, homologous TAIR8 CDS from *A. thaliana *and the position of the probe within the aligned sequences was used to assess whether the probe is likely to lie in coding or untranslated regions of the transcript. The results are summarised in Table 2. In most (eight) cases, the unigene aligns to an *A. thaliana *CDS and the position of the microarray probe can be inferred as being in a 3' UTR. In two cases, the alignment to an *A. thaliana *CDS suggests that the probe lies within the coding region.

**Table 2:** Position of probe sequence within unigenes aligned to *A. thaliana *CDS

**Unigene**	**BAC**	**Position of probe in BAC (bp)**	**Length unigene/bp**	**Arabidopsis CDS homologue**	**E value**	**Position of probe**
CD814561	KBrB005J17	29945 – 30004	555	AT1G67170	1.00E-24	3' UTR

EL590227	KBrH006E24	22739 – 22798	760	AT1G27595	3.00E-97	3' UTR

EV025509	KBrB013O20	3047 – 3106	606	AT3G06340	3.00E-41	Coding region

EV192260	KBrH066L21	23787 – 23846	720	AT5G29000	<1E-100	3' UTR

EX121951	KBrB026E08	69852 – 69911	649	AT2G33020	4.00E-87	3' UTR

JCVI_14643	KBrB091M07	67082 – 67141	998	AT4G38950	<1E-100	3' UTR

JCVI_23824	KBrB004L02	112948 – 113007	1100	AT5G19370	<1E-100	3' UTR

JCVI_32841	KBrB089M05	95567 – 95626	787	AT3G15920	<1E-100	Coding region

JCVI_39932	KBrH127P20	35513 – 35572	977	AT2G46220	<1E-100	3' UTR

JCVI_7760	KBrB048F07	71056 – 71115	2101	AT4G36390	<1E-100	3' UTR

Twelve unigenes were identified that had cognate genes in sequenced *B. rapa *BAC clones, but did not show homology to *A. thaliana *CDS. The sequences of these unigenes were assessed, using BLASTN, for similarity with any *A. thaliana *genomic sequences or other sequences in the NCBI nucleotide collection (nr/nt) database. The results are summarised in Table 3. In two cases, the unigene contains some sequences with homology to short stretches of *A. thaliana *genomic sequences. However, in most cases (ten), the unigenes appear to represent *Brassica*-specific sequence, as no similarities were identified with genomic sequences from *A. thaliana *or any other organism. The majority of these (eight) originate from positions in the *B. rapa *genome that lie between genes showing collinearity with the *A. thaliana *genome. The remaining two originate from positions within gene clusters (one of protein kinase-encoding genes and the other of oxidoreductase-encoding genes).

**Table 3:** Analysis of similarity of unigenes showing A genome-specific expression patterns and no similarity to *A. thaliana *CDS

**Unigene**	**Length unigene**	**Cognate BAC**	BLAST similarity to other organisms*	Genomic context**
EE447381	597	KBrB044C04	*A. thaliana *F6A14	Within protein-coding gene

EV084643	624	KBrB036M17	none	Between collinear conserved genes

EX052353	634	KBrB052E10	none	Between collinear conserved genes

EX117393	960	KBrB068E07	none	Within oxidoreductase gene cluster region

EX120283	685	KBrB080C12	none	Between collinear conserved genes

EX123623	634	KBrS011B08	none	Within kinase gene cluster region

EX133623	682	KBrB043M07	none	Between collinear conserved genes

EX140739	619	KBrB043B23	none	Between collinear conserved genes

JCVI_31720	1258	KBrH004B20	none	Between collinear conserved genes

JCVI_41745	490	KBrB043L02	*A. thaliana *F7O18	Within protein-coding gene

JCVI_6195	720	KBrH009D02	none	Between collinear conserved genes

JCVI_8626	708	KBrH125N23	none	Between collinear conserved genes

* E-value threshold < 1E-10

** "Collinear conserved genes" refers to genes of *B. rapa *and *A. thaliana *that show conserved synteny

## Discussion

We assembled unigenes using 810,254 EST sequences, mainly from three species: *B. napus*, *B. rapa *and *B. oleracea*. The assembly was conducted with the aim of co-assembling ESTs of orthologous genes (*i*ncluding homoeologue-pairs in *B. napus *from each of the A and C genomes), but resolving assemblies of paralogous genes (*i.e*. the genes related by the ancestral genome triplication observed in *Brassica *species). To do this, the assembly cut-off was set at 94% identity, based on our estimates of nucleotide conservation between paralogues of ~84% [13] and between A and C genome orthologues of 94–97% (unpublished). In total, 94,558 unigenes, representing 90,864 unique sequences were developed. An anticipated consequence of the close phylogenetic relationship between *Brassica *and *A. thaliana*, for which a complete genome sequence is available and has been annotated to a high standard, the majority of the unigenes (72,148) could be annotated and orientated on the basis of sequence similarity to proteins in the Uniprot100 database. The remaining 18,716 unigenes are candidates for encoding Brassica-specific proteins or non-coding RNAs.

In the absence of genomic sequence data, the functional significance of the large number of *Brassica*-specific unigenes is difficult to assess. As a first step, the assemblies were incorporated into the BAC sequence annotation for the *Brassica rapa *Genome Sequencing Project, enabling the identification of cognate genomic sequences for a proportion of the assemblies and contributing to the annotation of the emerging *B. rapa *genome sequence.

A 60-mer oligo microarray was developed using the unigene sequences and its utility validated by conducting an experiment aimed at testing its ability to analyse the transcriptomes of multiple *Brassica *species. Gene expression was analysed in two resynthesised *B. napus *lines and the *B. oleracea *and *B. rapa *lines used to produce them. The *B. napus *lines represented progeny resulting from both *B. oleracea *crossed onto *B. rapa *(thus possessing the *B. rapa *cytoplasm) and *B. rapa *crossed onto *B. oleracea *(thus possessing the *B. oleracea *cytoplasm). The 60-mer probe design enables an analysis of differential expression regardless of allelic variation due to SNPs or short indels which might interfere with transcript detection by the probes. The analysis showed that significant expression could consistently be detected in leaf tissue for 35,386 unigenes. This proportion of the total number of 94,558 unigenes (37.4%) is consistent with our expectations as many of the ESTs in the original collection were derived from other tissues (particularly developing seeds). Our criteria for significant expression were stringent (resulting in the elimination of 32,353 probes for which nevertheless at least one array detected significant expression). Expression was detected across all four genotypes for 27,355 unigenes (77.3% of those for which consistent expression was detected) and principal component analysis clearly resolved the individual microarray datasets for *B. rapa*, *B. oleracea *and resynthesised *B. napus*. Quantitative differences in expression were observed between the resynthesised *B. napus *lines for 98 unigenes, most of which could be classified into non-additive expression patterns, including 17 that showed cytoplasm-specific patterns.

In the two diploids, genome-specific expression patterns were observed for 7,851 unigenes (22.2% of those for which consistent expression was detected). These may represent instances in which the probes were designed to sequences that differ between the A and C genome orthologues. However, the anticipated sequence polymorphism rate between coding regions of orthologous genes of ~3.4% would typically result in ~2 differences per probe, which is unlikely to destabilize the hybridization sufficiently to abolish signal. We have, however, observed that sequences that are orthologous between the *Brassica *A and C genomes also differ in insertion-deletions (InDel) (unpublished), which could result in more extensive destabilization if overlapping the region to which the probe is designed. Alternatively, these may be sequences that are present in only one of the *Brassica *genomes, or their genome-specific expression may be tissue-dependent (we have analysed only leaf tissue). To begin to understand the basis for this difference, we exploited the emerging *B. rapa *genome sequences in order to characterize the genome sequences cognate to some of the unigenes showing genome-specific patterns of expression, as reported by the microarray. This revealed that, in the majority of cases, the probes are positioned in 3' UTR regions. However, ten of the aligned unigenes were found to be *Brassica*-specific sequences, including two that originate from complex loci comprising gene clusters. Therefore, we can hypothesise that a proportion of the unigenes showing genome-specific patterns of reported expression are likely to represent either *Brassica*-specific genes or *Brassica*-specific non-protein coding sequences. The observation of two instances of novel transcripts from clusters of genes that show evidence of recent duplication and rearrangements, and are reminiscent of some classes of disease resistance loci in plants, is particularly intriguing as it provides evidence for these loci producing novel genetic and transcriptional variation.

## Conclusion

We successfully developed and validated a microarray resource for use by the *Brassica *research community. The microarray enabled the detection of gene expression across all *Brassica *species tested for >27,000 unigenes. Genome-specific expression was observed for more than 7000 further unigenes. We anticipate that these will represent both species-specific transcripts and the consequences of variation of sequences within the regions of the unigenes represented by the array probes. Our studies demonstrated that the datasets obtained from the arrays can be used for typical analyses, including PCA and the analysis of differential expression. Our analysis of unigenes showing genome-specific expression patterns confirmed the transcription of sequences not represented in *A. thaliana*. Indeed, numerous transcripts were identified that represent *Brassica*-specific sequences. These transcripts would not be detectable using arrays designed with *A. thaliana *sequences and may represent functional genes not represented in other species.

## Methods

### Growth of plants

Seed was sown into Plantpak 9 cm pots containing Scotts Levington F1 compost (Scotts, Ipswich, UK) and covered with a plastic propagator lid. The seeds were germinated and grown in long day glass house conditions (16 hours photoperiod) at 15°C (400 W HQI metal halide lamps). Plants were pricked out after 11 days into Plantpak P15 modules containing Scotts Levington M2 compost and arranged into a four block randomised design with three plants each for each of the four genotypes per block and randomised within each block. Leaves were harvested 15 days after pricking out, 26 days after sowing. Leaf harvest was carried out as close to the midpoint of the light period as possible. The first true leaf of each plant was excised as close to the petiole as possible and the weight was recorded. Three leaf samples for each genotype from each experimental block were pooled and frozen in liquid nitrogen, giving a final harvest of four pooled leaf samples per genotype.

### Preparation of RNA

RNA was prepared by grinding tissue in liquid nitrogen and extracting using TRI Reagent (Sigma-Aldrich, St. Louis, MO, USA) according to the manufacturer's protocol. The RNA was resuspended in 50 μl DEPC treated water (Severn Biotech Ltd., Kidderminster, UK). The RNA samples were further purified using the Qiagen Mini Kit (Qiagen Inc., Valencia, CA, USA) according to the RNA Clean up protocol given in the RNeasy Mini Handbook (4^th ^edition, April 2006).

### Gene Expression Profiling

The quantity and purity of the extracted RNA was evaluated using a NanoDrop ND-1000 spectrophotometer (Nanodrop Technologies, Wilmington, DE, USA) and its integrity measured using an Agilent Bioanalyzer. For microarray hybridizations performed, 500 ng of total RNA from each sample was amplified and labeled with a fluorescent dye (Cy3) using the Low RNA Input Linear Amplification Labeling kit (Agilent Technologies, Palo Alto, CA, USA) following the manufacturer's protocol. The amount and quality of the fluorescently labeled cRNA was assessed using a NanoDrop ND-1000 spectrophotometer and an Agilent Bioanalyzer. A consistent amount of Cy3-labeled cRNA (1.6 μg) were hybridized to the custom Brassica microarray, which was manufactured by Agilent Technologies, for 17 hours, prior to washing and scanning. Data were extracted from scanned images using Agilent's Feature Extraction Software (Agilent Technologies).

### Data Analysis

Gene expression data was loaded into the Rosetta Resolver^® ^Gene Expression Analysis System version 7.0.0.1.9 and biological replicates were combined using an error-weighted average. Ratios were then calculated comparing each possible combination of samples. The criteria for identification of differentially expressed transcripts was an absolute fold change value > 2.0, a log ratio p-value < 0.001, and a log(10) intensity measurement > -1.8. Rosetta Resolver was used to perform a principal component analysis (PCA) using z-score transformed intensity data for all non-control features present on the microarray for each of the 16 samples that were profiled.

The statistical significance of probes representing differentially expressed transcripts was determined using the Bayesian-moderated test statistic described in [22]. The statistic was calculated in a linear model framework provided by the library limma, which is part of the BioConductor suite of libraries for the statistical programming language R. The *p*-value cut-off, given above, for significance was established by inspecting the distribution of *p*-values associated with the control probes on the microarray.

### Annotation and databases

Finished *Brassica rapa *BAC sequences available in the public domain were annotated using the Brassica 95 k unigene set as described below and the results published to complement the other annotation tracks available through the GBrowse genome browser at . Briefly, the 95 k set was first queried against each BAC sequence using BLASTN 2.0MP-WashU [20-Apr-2005] [23] implemented on a Linux cluster with an initial E-value threshold parameter of 1 × 10^-50^. Positive hits were saved and the corresponding transcript assemblies were then re-aligned against the genomic sequence with BLAT [24] using a sequence identity threshold of 95%. Coordinates of the BLAT alignment blocks were parsed to GFF format with the annotation Perl script and loaded into the MySQL database driving the Genome browser, which is also directly accessible via a programmatic interface to allow querying.

In addition, full details of the composition of the 95 k unigene set were loaded into a separate MySQL database which can be interrogated through a web front-end also at . This database may be searched with text terms or fragments (which will be wild-carded) for matches on a number of fields, including assembly or singleton identifier, the identifier, gene name, description or source organism of the best UniProt BLASTX hit and, where appropriate, the identifiers, tissue sources and source Brassica species of the ESTs contributing to an assembly. Search results are returned in HTML tabular form and, where appropriate, are marked up with hyperlinks to GBrowse views, EBI sequence and InterPro descriptions and NCBI dbEST records. The sequence of the unigene is also returned and, if it appears on the array, the 60-mer Agilent probe designed is rendered in lower case.

Finally, the DNA sequences of all members of the 95 k unigene set are available for similarity matching through a BLAST server at  and the fasta sequence file is downloadable from the FTP site .

## Competing interests

The authors declare that they have no competing interests.

## Authors' contributions

IB conceived of the study, participated in its design and coordination, and helped to draft the manuscript. MT and ND conceived and implemented the BAC annotation and assembly database and helped to draft the manuscript. FF grew the plants and prepared the RNA. EKL participated in the design of the microarray, helped formulate the experimental design and the drafting of the manuscript. PH participated in the design of the microarray. FC and CT performed the EST assembly and analysis and supplied the output files for microarray design. AM performed statistical computing on the output files, including exploratory analysis and statistical inference of the significant differential transcriptional abundance. All authors read and approved the final manuscript.

## Supplementary Material

Additional file 1 **Spreadsheet 1**. Unigenes for which probes report significant (P < 0.001) differences between expression levels in *B. napus *1 and *B. napus *2 Click here for file

Additional file 2 **Spreadsheet 2**. Classification of qualitative expression patterns reported for unigenes Click here for file

Additional file 3 **Spreadsheet 3**. Classification of expression patterns of unigenes for which probes report significant (P < 0.001) differences between expression levels in *B. napus *1 and B. *napus *2. Definition of classification terms; *non-additive*: expression in one or both *B. napus *lines departs from that expected for additive expression of the values observed in the parent lines; *cytoplasm-specific*: expression in *B. napus *matches the characteristics of that in the maternal parent line; *unclassified*: insufficient data are available to permit classification. The small variation in intensity values reported for a given genotype arises from normalizations being performed independently for each pairwise comparison conducted. Click here for file
